# Long branch attraction, taxon sampling, and the earliest angiosperms: *Amborella *or monocots?

**DOI:** 10.1186/1471-2148-4-35

**Published:** 2004-09-28

**Authors:** Saša Stefanović, Danny W Rice, Jeffrey D Palmer

**Affiliations:** 1 Department of Biology, Indiana University, Bloomington, IN 47405, USA; 2 Department of Biology, University of Toronto at Mississauga, Mississauga ON, L5L 1C6, Canada

## Abstract

**Background:**

Numerous studies, using in aggregate some 28 genes, have achieved a consensus in recognizing three groups of plants, including *Amborella*, as comprising the basal-most grade of all other angiosperms. A major exception is the recent study by Goremykin et al. (2003; *Mol. Biol. Evol*. 20:1499–1505), whose analyses of 61 genes from 13 sequenced chloroplast genomes of land plants nearly always found 100% support for monocots as the deepest angiosperms relative to *Amborella*, *Calycanthus*, and eudicots. We hypothesized that this conflict reflects a misrooting of angiosperms resulting from inadequate taxon sampling, inappropriate phylogenetic methodology, and rapid evolution in the grass lineage used to represent monocots.

**Results:**

We used two main approaches to test this hypothesis. First, we sequenced a large number of chloroplast genes from the monocot *Acorus *and added these plus previously sequenced *Acorus *genes to the Goremykin et al. (2003) dataset in order to explore the effects of altered monocot sampling under the same analytical conditions used in their study. With *Acorus *alone representing monocots, strongly supported *Amborella*-sister trees were obtained in all maximum likelihood and parsimony analyses, and in some distance-based analyses. Trees with both *Acorus *and grasses gave either a well-supported *Amborella*-sister topology or else a highly unlikely topology with 100% support for grasses-sister and paraphyly of monocots (i.e., *Acorus *sister to "dicots" rather than to grasses). Second, we reanalyzed the Goremykin et al. (2003) dataset focusing on methods designed to account for rate heterogeneity. These analyses supported an *Amborella*-sister hypothesis, with bootstrap support values often conflicting strongly with cognate analyses performed without allowing for rate heterogeneity. In addition, we carried out a limited set of analyses that included the chloroplast genome of *Nymphaea*, whose position as a basal angiosperm was also, and very recently, challenged.

**Conclusions:**

These analyses show that *Amborella *(or *Amborella *plus *Nymphaea*), but not monocots, is the sister group of all other angiosperms among this limited set of taxa and that the grasses-sister topology is a long-branch-attraction artifact leading to incorrect rooting of angiosperms. These results highlight the danger of having lots of characters but too few and, especially, molecularly divergent taxa, a situation long recognized as potentially producing strongly misleading molecular trees. They also emphasize the importance in phylogenetic analysis of using appropriate evolutionary models.

## Background

**Figure 1:**
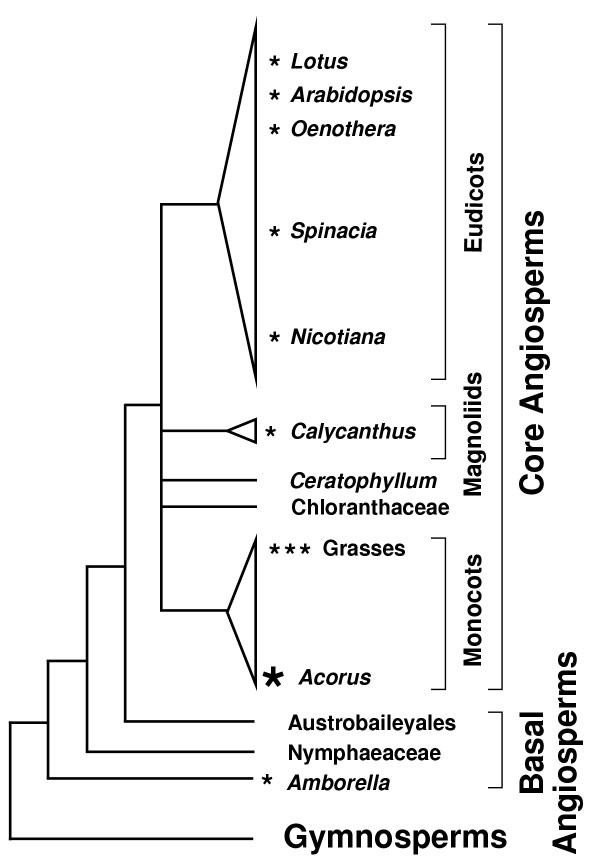
**Current consensus hypothesis of angiosperm relationships. **Tree topology is based on [42, 91] and references in Table 1. Small asterisks indicate the general phylogenetic position of the ten angiosperms (generic names shown for all but the three grasses) examined by Goremykin et al. [19]. The large asterisk indicates the addition in this study of the early-arising monocot *Acorus *to the Goremykin et al. [19] dataset. The height of the triangles reflects the relative number of species in eudicots (~175,000 species), monocots (~70,000), and magnoliids (~9,000) as estimated by Judd et al. [18] and Walter Judd (personal communication). The other five angiosperm groups shown contain only between 1 and ~100 species.

A correct understanding of relationships among the "earliest" lineages of angiosperms is important if we wish to elucidate the causes and consequences of their origin, to understand patterns and tempos of character evolution in the earliest lineages, and to decipher subsequent patterns of diversification. [We sometimes use "earliest", "deepest", "basal", etc. as a convenient shorthand to refer to plants hypothesized to belong to lineages that result from the first or one of the first evolutionary branchings within angiosperm evolution. We do not mean to imply that any extant plants (e.g., *Amborella*) are themselves the "earliest" angiosperms, but rather that they belong to the lineage of angiosperms that resulted from the first evolutionary split in angiosperm evolution. When the term "sister" is used to refer to a phylogenetic placement it refers to the sister group to the rest of the angiosperms unless otherwise specified.] A breakthrough in the seemingly intractable problem of identifying the earliest lineages of angiosperms occurred in 1999 and 2000, when each of many multigene studies identified the same three groups as the earliest branching angiosperms [1-9]. Most of these studies, as well as most subsequent analyses [10-17] have converged on the placement of the monotypic genus *Amborella*, a vessel-less shrub with unisexual flowers endemic to New Caledonia, as the sister-group to all living angiosperms (Fig. 1, Table 1), with the next two divergences within angiosperms corresponding to the water lilies (Nymphaeaceae) and then the Austrobaileyales. This grade leads toward the well-supported remainder of the flowering plants, also known as core angiosperms [18] (Fig. 1). The monophyly of each of the five lineages of core angiosperms is well established, but relationships among them are unclear (Fig. 1).

**Table 1:** Comparison of recent studies^a ^that identify the sister lineages of angiosperms.

Study reference	No. of genes (genomes^b^)	No. of angiosperms	No. of nucleotides	*Amborella *sister to the rest of angiosperms^c^		Basal vs. core angiosperms^c^		Monophyly of monocots^c^	
[4]	5 (c, m, n)	97	8,733	+	90	+	97	+	99/98
[3]	5 (c, m, n)	45	6,564	+	94^d^	+	99^d^	+	98^d^
[6]	3 (c, n)	553	4,733	+	65^e^	+	71^e^	+	95^e^
[1]	2 (n)	26	2,208	+	92/83^f^	+	86	+	100
[2]	2 (n)	52	2,606	+	88/57^f^	+	68	+	87
[8]	6 (c, m, n)	33	8,911	-	n/a^g^	+	99	+	100
[9]	17 (c)	18	14,244	+	69	+	94	+	53
[11]	1 (c)	38	4,707	+	99	+	100	+	100
[14]	1 (c)	361	1,749	+	86	+	89	+	99

^a^Not included are several other studies also supportive of *Amborella*-sister, but which are largely duplicative of the above [5, 7, 31], or whose structure does not match sufficiently with the structure of this table [10, 12, 13], or which have extremely limited sampling (6 taxa) within angiosperms [15].

^b^c = chloroplast; m = mitochondrial; n = nuclear

^c^Indicated relationship recovered (+) or not recovered (-); parsimony BS values shown unless otherwise specified. See Fig. 1 for definition of indicated relationships.

^d^Only BS values derived from ML analysis are shown.

^e^Jackknife support values.

^f^Bootstrap values were inferred from separate *phyA *and *phyC *treatments; other BS values in this study were derived from concatenated *phyA *and *phyC *sequences.

^*g*^n/a – not applicable. This study found *Amborella*+*Nymphaea *as sister to all other angiosperms (see Discussion).

In sharp contrast stands the study of Goremykin et al. [19], in which the *Amborella *chloroplast genome was sequenced and in which 61 protein genes shared among 13 land plants (including 10 angiosperms) were analyzed. In 31 of 33 phylogenetic analyses this study found that "*Amborella *is not the basal angiosperm and not even the deepest branching among dicots" ([19] Abstract). Instead, these results indicate, with 100% BS in most analyses, that the first split within angiosperm evolution occurred between monocots and dicots. Goremykin et al. [19] imply that the earlier studies are in error with respect to the placement of *Amborella *because these "studies were based on a limited number of characters derived from only a few genes" and used "unmasked sequences of chloroplast genes [i.e., with all three codon positions included] with high substitution rates at their synonymous sites" (p. 1503).

Thus, we are faced with a major paradox. On the one hand, many different studies, employing in aggregate 28 different genes (19 chloroplast, five mitochondrial, and four nuclear; Table 1), consistently and strongly place the branch leading to *Amborella *deeper in angiosperm evolution than the branch leading to the monocots, whereas a study that employed twice as many genes found the opposite result, also with strong support. It is critical to resolve this paradox, for the groups and issues involved are such important ones in angiosperm phylogeny.

One notable difference between the two sets of studies concerns taxon sampling, which can be critical in phylogenetic analysis [20-24]. Even though sampling strategies in the *Amborella*-deep studies listed in Table 1 varied substantially, ranging from 18 to 553 species of angiosperms and from 2,208 to 14,244 nucleotides (NT) of aligned data, a commonality was their relatively broad taxon sampling. Most of these studies attempted to represent the diversity of living angiosperms by including critical species identified by prior morphological [25-28] and single-gene molecular analyses [29-31]. Even the listed study with the fewest taxa [9] was based on exemplar species, compiled by the Green Plant Phylogeny Research Coordination Group and chosen to represent most of the major putatively basal lineages suggested by a large body of previously accumulated results. In contrast, the Goremykin et al. [19] study included only 10 angiosperms. Five of these belong to a single derived group (eudicots) and three are grasses (the only monocots sampled), leaving *Amborella *and *Calycanthus *(the only sampled member of the other three lineages of core angiosperms) as the other two angiosperms sampled (Fig. 1). It is known that grasses have accelerated substitution rates in all three genomes [9,32-35], especially the chloroplast genome, making them a poor representative for such a large and diverse group as monocots.

Relevant here is that the grasses-sister topology obtained by Goremykin et al. [19] (see their Fig. 3, which also corresponds to our Fig. 3A) shows one long branch, leading to grasses, connecting to another long branch, separating angiosperms from the outgroups. When the outgroups are removed and the Goremykin et al. [19] tree is taken as an unrooted network, it becomes apparent that there is no difference between their ingroup topology and those of studies that obtained the *Amborella*-sister rooting. In other words, given the taxonomic sampling of Goremykin et al. [19], their grasses-sister topology differs from the canonical *Amborella*-sister topology only with respect to where the outgroup branch attaches [36], either to grasses or to *Amborella *(see Discussion and Fig. 8 for an elaboration of this point).

These observations led us to suspect that the grasses-sister topology is an artifact stemming from long branch attraction (LBA), a phenomenon known [37-39] to give strongly supported, but spurious results under precisely the set of conditions operative in the Goremykin et al. [19] study. These are 1) inadequate taxon sampling, 2) large amounts of data per taxon, 3) two known long branches (the grass branch and the outgroup branch) separated by short internodes, and 4) phylogenetic analyses that do not account for rate heterogeneity.

The current study was undertaken to test whether the grasses-sister topology is indeed an LBA artifact. We hypothesize that, by analyzing the Goremykin et al. [19] dataset with a focus on rate heterogeneity and taxon sampling of monocots, the *Amborella*-sister topology will be recovered instead. In addition, we carried out a similar, but much more limited set of analyses in response to a follow-up paper by Goremykin et al. [40] that appeared while this manuscript was in the final stages of preparation and which similarly challenged the position of *Nymphaea *as a basal angiosperm.

## Results

### Addition of *Acorus*

We gathered new sequence data for an additional monocot representative, *Acorus*, and added it to the 13 taxa, 61 gene first- and second-position alignment matrix of Goremykin et al. [19] to give a 14 taxa, 61 gene first- and second-position alignment matrix. *Acorus *was chosen for two reasons. First, it is well supported as the sister to all other monocots [41-43]. Thus, *Acorus *plus grasses represent monocot diversity about as well as any two groups of monocots. Second, unlike grasses, its chloroplast genome does not appear to have evolved at unusually high rates [9,44]. The *Acorus *dataset consisted of 40 protein gene sequences, 22 newly determined in this study and 18 from preexisting databases. This corresponds to 65.6% (40/61) of the genes and 71.4% (32,072/44,937) of the nucleotide characters analyzed by Goremykin et al. [19].

A number of initial analyses were conducted in parallel on the "full" *Acorus *matrix, containing data for all 61 genes and including gaps where data for *Acorus *were not available, and a "truncated" matrix, containing only those 40 genes where *Acorus *sequences were available. Inspection of the resulting trees revealed no topological incongruences and no significant change in bootstrap support (BS) between the full and truncated analyses [see Additional files 1 and 2]. The results presented hereafter for *Acorus *are based on the full matrix dataset. This allows us to include all available relevant data, allowing the fullest and most direct comparisons to the Goremykin et al. [19] analyses.

**Figure 2:**
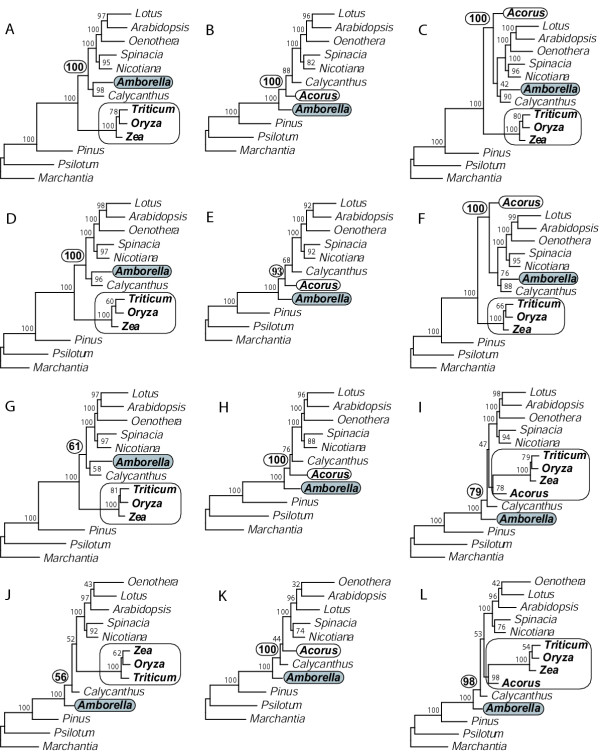
**The effect of changing sampling of monocots as a function ofphylogenetic method. **Analysis of the 61-gene data matrix using: Rows **A-C**, DNA parsimony; **D-F**, protein parsimony; **G-I **DNA ML HKY85 with no rate categories; **J-L**, RY-coded DNA parsimony. The first column of trees is with the Goremykin et al. [19] taxon sampling (grasses, but not *Acorus*), the second is with *Acorus *but not grasses, and the third is with both grasses and *Acorus*. All analyses used the first- and second-position matrix, either with or without the addition of *Acorus *as explained in Methods. Trees **J-L **use the same matrices, but with the nucleotides RY-coded.

Representative results of either adding *Acorus *to the Goremykin et al. [19] matrix or substituting it for grasses are shown in Fig. 2. Using *Acorus *instead of grasses to represent monocots has a major effect on the results. This is especially dramatic for equal-weighted maximum parsimony (MP) analyses of both nucleotides and amino acids, where there is a shift from 100% BS for monocots-sister when only grasses are used to represent monocots (Figs. 2A and 2D) to 100% and 93% support for *Amborella*-sister when *Acorus *is used instead (Figs. 2B and 2E). The same topological shift is seen with maximum likelihood (ML) using equal rates across sites (cf. Figs. 2G and 2H), although the swing in BS values is less pronounced (61% for grasses-sister vs. 100% for *Amborella*-sister). Transversion parsimony (RY-coding) of the original dataset (Fig. 2J) gives the *Amborella*-sister topology, but with poor support (56%). Substituting *Acorus *for grasses improves the support for *Amborella*-sister to 100% (Fig. 2K).

**Figure 3:**
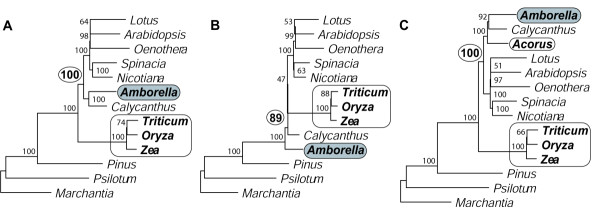
**Neighbor joining analyses using different evolutionary models and/or taxon sampling. **Distance matrices were calculated from the first- and second-position matrix of Goremykin et al. [19] using (**A**) the K2P model, (**B**) the ML HKY85 model with four gamma-distributed rate categories and parameters estimated from the corresponding ML analysis, and (**C**) the K2P model with *Acorus *added to the first- and second-position matrix as described in Methods.

Inclusion of both grasses and *Acorus *produced two very different topologies, depending on the method used. On the one hand, standard MP, with both nucleotides (Fig. 2C) and amino acids (Fig. 2F), gives a grasses-sister topology in which monocots are paraphyletic with 100% BS (i.e., there is 100% support for *Acorus *as the sister to "dicots" to the exclusion of grasses). On the other hand, equal-rates ML (Fig. 2I) and transversion parsimony (Fig. 2L) give an *Amborella*-sister topology, with moderate (79%) to strong (98%) support, in which monocots are monophyletic with equivalent support.

To make the results more directly comparable to the Goremykin et al. study [19] and to investigate the performance of various distance-based models, we tested many different neighbor joining (NJ) models. We did this also because, of all MP, ML and NJ methods initially investigated, the only approaches that failed to give the *Amborella*-sister topology when *Acorus *was substituted for grasses were the NJ methods without a ML model. When the PAUP* [45] distance is set to any of 12 settings (Mean, P, JC [46], F81 [47], TajNei [48], K2P [49], F84 [50], HKY85 [51], K3P [52], TamNei [53], GTR [54,55] or LogDet [56,57]), *Amborella*, *Calycanthus*, and *Acorus *form a monophyletic group with 100% BS. Importantly, however, this same grouping is obtained, with all 12 distance settings, even when grasses are included, such that, as in equal-weighted parsimony analyses (Figs. 2C and 2F), grasses are sister to all other angiosperms and monocots are not monophyletic (Fig. 3C and analyses not shown).

Finally, it should be noted that ML and NJ methods using models (see next section) that give *Amborella*-sister when only grasses represent monocots, continue to do so, but with higher BS, when *Acorus *is added, either with or without grasses [see Additional files 1 and 2].

### Site-to-site rate heterogeneity

If the lineage leading to *Amborella *is sister to the rest of angiosperms, as the analyses with *Acorus *strongly indicate, why do so many of the Goremykin et al. [19] analyses support the grasses-sister topology? We explored this question by conducting analyses using a broad range of models and methods as applied to their data matrix (i.e., with only grasses representing monocots).

We first compared the relative likelihood of the grasses-sister and *Amborella*-sister topologies using ML with all 56 combinations of the 14 substitution models and four rate-heterogeneity conditions specified by the MODELBLOCK script provided by MODELTEST [58]. The four rate-heterogeneity conditions are 1) equal rates across sites, 2) estimated percentage of invariant sites, 3) four gamma-distributed rate categories and 4) a combination of invariant sites and gamma-rate categories. With equal rates across sites, the grasses-sister topology received the higher likelihood for all 14 substitution models (Table 2). For the least complex, Jukes-Cantor [46] model (a single substitution rate with equal base frequencies), all four rate-heterogeneity conditions preferred the grasses-sister topology. In a more complex model (F81), which uses estimated base frequencies, the *Amborella-*sister topology was preferred when either invariant sites or gamma rate categories were used but not when they were used in combination. For the other 12 models, the *Amborella-*sister topology was preferred for all three conditions that allowed for rate heterogeneity across sites (Table 2).

**Table 2:** The 56 MODELTEST models and the grasses- or *Amborella*-sister topology that received the higher likelihood.

**Model**	**equal**	**+I**	**+G**	**+I +G**
**JC**	grasses	grasses	grasses	grasses
**F81**	grasses	Amborella	Amborella	grasses
**K80**	grasses	Amborella	Amborella	Amborella
**HKY**	grasses	Amborella	Amborella	Amborella
**TrNef**	grasses	Amborella	Amborella	Amborella
**TrN**	grasses	Amborella	Amborella	Amborella
**K81**	grasses	Amborella	Amborella	Amborella
**K81uf**	grasses	Amborella	Amborella	Amborella
**TIMef**	grasses	Amborella	Amborella	Amborella
**TIM**	grasses	Amborella	Amborella	Amborella
**TVMef**	grasses	Amborella	Amborella	Amborella
**TVM**	grasses	Amborella	Amborella	Amborella
**SYM**	grasses	Amborella	Amborella	Amborella
**GTR**	grasses	Amborella	Amborella	Amborella

The four rate-heterogeneity conditions used in these MODELTEST analyses are: 1) "equal" = equal rates across sites; 2) "+I" = estimated percentage of invariant sites; 3) "+G" = four gamma-distributed rate categories; and 4) "+I+G" = combination of invariant sites and 4 gamma-rate categories.

These results held when the parameters estimated on one topology (either *Amborella*- or grasses- sister) were used to calculate the likelihood of the other topology (the topology used had only a minor effect on the values of the parameter estimates). For both topologies, the model chosen by MODELTEST using either the hierarchical likelihood ratio tests or the Akaike information criterion was the 5-substitution-type-transversion (TVM) + I + G model, where the probability of going between A and G is equal to that of C and T. With this model, using parameter estimates from either topology, a heuristic search found the *Amborella*-sister topology with 98% BS, and the SH-test [59] showed the grasses-sister topology to be significantly worse at the 5% level (p = 0.04).

**Figure 4:**
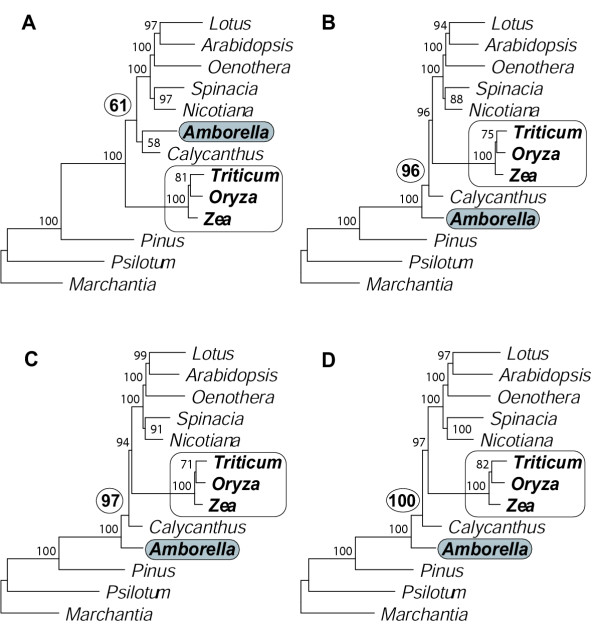
**Maximum likelihood analyses using different evolutionary models. **Trees **A-C **were calculated using the first- and second-position Goremykin et al. [19] matrix. Tree **D **was calculated using all three codon positions. All trees were built using ML with the HKY85 model and the following treatments of rate heterogeneity: **A**. No rate categories. **B**. Four gamma-distributed rate categories. **C**. Estimated proportion of invariant sites (no gamma rate categories). **D**. No rate categories (all three positions). Parameters were estimated separately for each analysis as described in Methods.

These MODELTEST analyses identified site-to-site rate heterogeneity, accounted for using either gamma-distributed rates or invariant sites, as a critical analytical parameter. We therefore explored this in greater detail using one particular substitution model, the HKY85 model [51]. We chose the moderately complex and commonly used HKY85 substitution model with empirical base frequencies over the TVM model to help speed up the calculation of bootstrap replicates. A ML-HKY85 analysis with equal rates and an estimated transition:transversion (Ti/Tv) ratio of 1.485 gives the same, grasses-sister topology (Fig. 4A) as found by Goremykin et al. [19] (see Fig. 2G, which is equivalent topologically to their Fig. 3), albeit with low BS (61%) for grasses-sister. In contrast, a tree built using four rate categories, with the gamma shape parameter (α = 0.31) estimated from the Goremykin et al. [19] matrix and topology, gives 96% BS for *Amborella*-sister (Fig. 4B). Although we present here only the commonly used, four-rate-category model, a two-rate-category model gives the same qualitative results in all cases analyzed [see Additional file 3].

**Figure 5:**
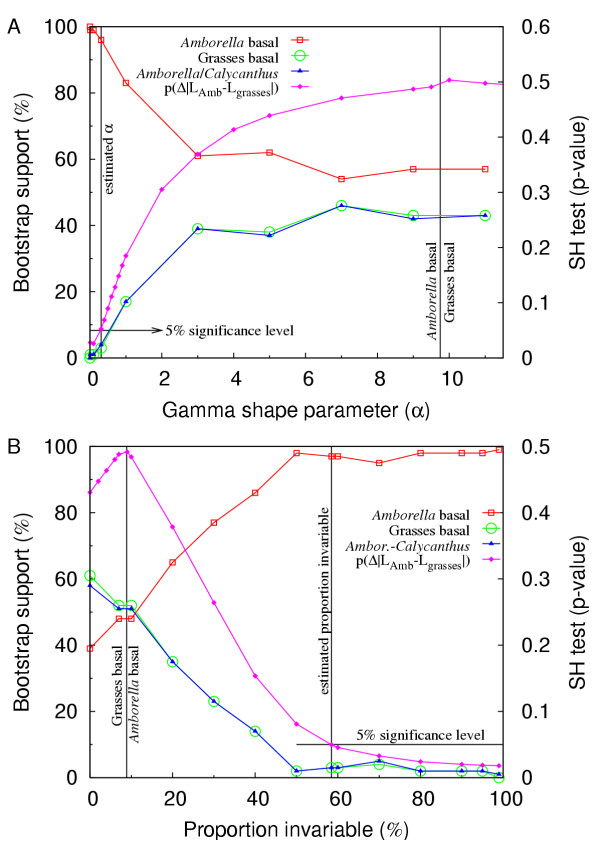
**Bootstrap support and the SH-test p-value for the *Amborella*-sister or grasses-sister topologies as a function of (A) the gamma distribution α parameter value or (B) the proportion of invariable sites. **The left vertical line in A and right line in B indicate the rate-heterogeneity parameter estimated from the data. The right vertical line in A and left line in B indicate the boundary where the topology of the best tree transitions between *Amborella*-sister and grasses-sister. All analyses were performed using the 61-gene first- and second-position matrix of Goremykin et al. [19] and the ML HKY85 model with the α parameter or proportion of invariant sites indicated on the X-axis. The transition-transversion parameter was estimated for each specified rate-heterogeneity parameter. p(Δ|L_Amb_-L_grasses_|) signifies the SH-test p-value for the difference between the likelihood scores of the two topologies. Bootstrap searches and SH-tests were performed as described in Methods.

To assess the stability of the topology to changes in the α parameter, we scanned the range α = [0.01–20.0], with the number of rate categories fixed at four. The same, *Amborella*-sister topology obtained using the estimated α (0.31) was also recovered over a wide range of α values (α = 0.01–9.0; Fig. 5A). The BS for *Amborella*-sister and the SH-test p-value [59] of the *Amborella*-sister over the grasses-sister topology both improve as α decreases to the estimated value and continue to improve as α approaches zero (Fig. 5A). As α approaches infinity, the rate categories approach the same value (i.e., equal rates) [60]. Accordingly, the BS and p-value curves in Fig. 5 approach the values of the equal-rates trees.

We performed a similar analysis with the proportion of invariant sites (Plnvar option in PAUP). Using the estimated PInvar = 0.58 without gamma-distributed rate categories, we obtained the *Amborella*-sister topology (Fig. 4C) with 97% BS. As with α, the *Amborella*-sister topology was stable over a wide range of PInvar [0.09 <= PInvar <= 0.995 (Fig. 5B)]. The BS and the SH-test p-value for *Amborella*-sister improve as PInvar increases (Fig. 5B). The SH-test for *Amborella*-sister is significant at the 5% level using the estimated value of PInvar and remains significant as PInvar increases.

The BS for a sister-group relationship of *Amborella *and *Calycanthus *is identical (within the variance expected for BS values) with that for grasses-sister across the entire range of both α and PInvar values, while both of these BS values always equal 100 minus the BS value for *Amborella*-sister (Figs. 5A and 5B). This is exactly as expected (see Discussion) if the only difference between the grasses-sister/*Amborella*+*Calycanthus *topology and the *Amborella*-sister topology is where the outgroup branch roots within angiosperms. Put another way, almost all of the BS replicates were one of these two topologies.

There are 20,071 (out of 30,017; 66.9%) constant sites in theGoremykin et al. [19] matrix. When these constant sites are removed, the highest HKY85 ML tree (using equal rates) places *Amborella*-sister with 98% BS and with p = 0.03 for the SH-test relative to grasses-sister [see Additional file 4, Fig. A]. Furthermore, NJ analysis with the equal-rate ML model also obtains *Amborella*-sister (with 100% BS) when constant sites are removed [see Additional file 4, Fig. B]. This is another way of allowing the rates to increase since the rates of the sites that are changing are not constrained by the constant sites. This allows the ML model to work with a more homogenous set of rates and reduces the need for using rate categories. Removing these constant sites allows the ML model to simulate the actual evolutionary process of sites that are changing more accurately than when imposing a proportion of invariant sites because there is no invariant site weighting of the sites that are changing. As a consequence of the faster rate with constant sites excluded, the branch lengths of the resulting trees are ~2.6 times longer than when constant sites are included.

We further explored the NJ method using ML models of evolution to compute distances and with constant sites included. We were able to precisely reproduce the grasses-sister result (Fig. 3 from Goremykin et al. [19]) with NJ and the K2P model(Fig. 3A). NJ using a distance matrix calculated based on ML and using parameters estimated with the HKY85 model with equal rates alsogives grasses-sister with 100% BS. However, distances calculated using the ML HKY85 model and estimated proportion of invariant sites puts *Amborella-*sister with low BS of 58% [see Additional file 5], while distances derived from the ML HKY85 model with four gamma-distributed rate categories estimated gives *Amborella*-sister with stronger support (89%; Fig. 3B).

### Third codon positions

In order to most directly assess the Goremykin et al. [19] analyses, which used only first and second codon position, the above analyses were restricted to first and second codon positions. In addition, however, most of the above analyses were also carried out with a dataset that includes all three codon positions. The resulting trees provide similar if not higher support for *Amborella*-sister than those obtained with just first and second positions. For example, using all three positions, the gamma rates ML tree analogous to Fig. 4B gives 100% BS for *Amborella*-sister, and the ML distance based NJ tree analogous to Fig. 3B gives 99% BS for *Amborella*-sister (trees available upon request). The most noteworthy shift towards stronger support involves ML analysis with equal rates, where inclusion of third positions changes the topology, from grasses-sister (with 61% BS; Fig. 4A) to *Amborella*-sister (and with 100% support; Fig. 4D). We also conducted a few analyses of third positions only (again using the set of taxa analyzed by Goremykin et al. [19]). These too recovered *Amborella*-sister, with 100% BS using ML with either equal rates or gamma-distributed rates [see Additional file 6].

### Individual gene analyses

**Figure 6:**
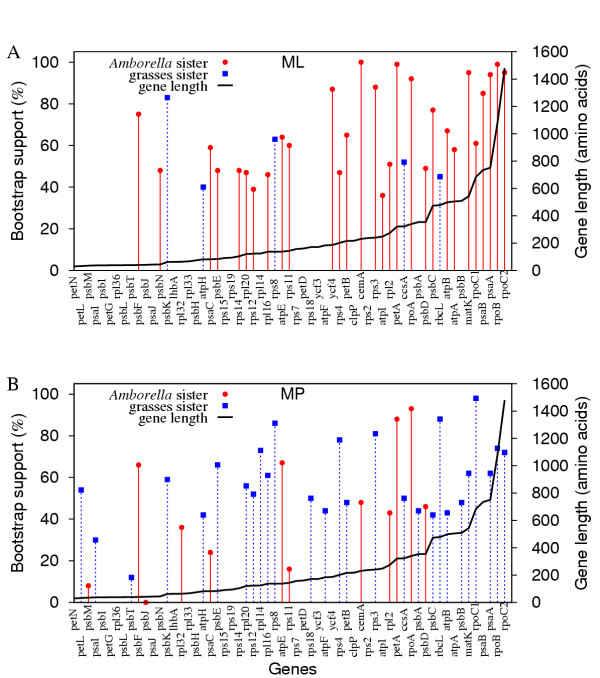
**Support for *Amborella*-sister or grasses-sister from the 61 chloroplast genes analyzed individually. A**. ML HKY85 analyses with four gamma-distributed rate categories. Parameter estimates were calculated individually for each gene in a manner analogous to that performed on the concatenated dataset. **B**. MP analyses. All three codon positions are included in all analyses shown in both figures. Solid red lines correspond to *Amborella*-sister and dashed blue lines to grasses-sister topologies.

By taking rate heterogeneity into account or improving taxon sampling, we have shown that the concatenated genes dataset supports the *Amborella*-sister hypothesis, strongly so in most analyses. To explore the effects of phylogenetic methods and taxon sampling on individual gene analyses, we analyzed each of the 61 genes in the Goremykin et al. [19] dataset individually (Fig. 6). These much smaller subsets of data are, as expected, more sensitive than the concatenated dataset to the model of DNA evolution, taxon sampling, and inclusion/exclusion of third positions. Without appropriately taking these factors into account some genes give topologies that conflict with the current consensus view of plant phylogeny. With all three positions and using ML with four gamma-distributed rate categories, the highest likelihood tree in 29 of 61 genes is the *Amborella*-sister topology and only five genes give grasses-sister (Fig. 6A). The highest scoring trees for the remaining genes (most of which are short) place a wide variety of groups as sister, in nearly all cases with low BS (data not shown). Bootstrap support values and the number of trees having *Amborella *sister increase with gene length (Fig. 6A). When MP is used on the same datasets the opposite pattern is observed. Here, the grasses are sister in 27 of 61 trees, whereas *Amborella *is sister with only 12 genes (Fig. 6B). Excluding third positions results in the same trend in terms of MP versus ML, but the support values are much lower and the number of highly unlikely topologies is much higher (see Additional file 7).

The single gene trees also illustrate the effect of taxon sampling. When *Acorus *is added and all three positions are used in ML analyses with four rate categories, none of the gene trees find monocots sister, whereas exactly half of the 40 genes put *Amborella *sister [see Additional file 8, top figure]. When the third position is excluded, 12 genes put *Amborella *sister and BS levels drop significantly, while still no genes put monocots sister [see Additional file 8, bottom figure]. Very similar results are obtained when the grasses are removed [see Additional file 9]. In contrast to the parsimony results without *Acorus *(where grasses-sister is the favored topology; Fig. 6B), when *Acorus *is added and parsimony is used (with all three positions), only two genes put monocots sister (and both with low, 13 and 34%, BS), whereas 11 of 40 genes put *Amborella *sister [see Additional file 10, top figure]. With *Acorus *added and grasses removed, 21 genes place *Amborella *sister and 1 places *Acorus *sister [see Additional file 10, bottom figure].

### Addition of Nymphaea

While this manuscript was in the final stages of preparation, the chloroplast genome sequence of *Nymphaea alba *became available (released to EMBL database on July 13, 2004). This sequence was generated as part of a very recent study, also by Goremykin et al. [40], in which it was added, as the only new sequence, to the same data matrix as analyzed in their earlier study [19] and subjected to a similar set of phylogenetic analyses. Under these conditions, the grasses-sister topology was again recovered (and with 100% support) in nearly all analyses, with *Nymphaea *and *Amborella *recovered as sister taxa (also with 100% support). In their abstract, Goremykin et al. [40] present these findings as supporting their prior conclusion [19] that monocots are sister to the rest of angiosperms. However, their Discussion presents a more nuanced treatment than before, concluding that "we may be some ways from being confident of identifying the most basal angiosperms. Clearly the sequencing of genomes for more closely related outgroups and putatively basal angiosperms will be important for overcoming potential problems of model misspecification and long-branch attraction."

**Figure 7:**
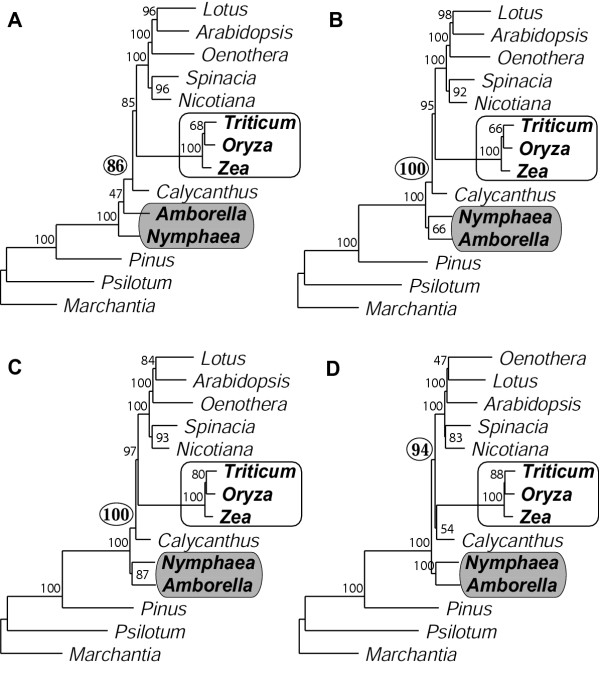
**Inclusion of *Nymphaea *in analyses that account for rate heterogeneity. A**. ML HKY85 with no rate categories (cf. Fig. 4A). **B**. ML HYK85 with four gamma-distributed rate categories (cf. Fig. 4B). **C**. ML with estimated proportion of invariant sites (no gamma rate categories; cf. Fig. 4C). **D**. NJ using a ML HKY85 model with four gamma-distributed rate categories to calculate distances (cf. Fig. 3B). All analyses used first- and second-positions only.

We carried out a limited set of analyses of the 14-taxa Goremykin et al. [40] data matrix. We did so because of time constraints and because it became immediately clear from our relatively few analyses with *Nymphaea *that our main results and conclusions were entirely unchanged by its inclusion/exclusion. Using the Goremykin et al. [40] methods, we also recovered the same, grasses-sister trees they reported (data not shown). However, when using analytical conditions described in the preceding sections, we never found grasses-sister (Fig. 7). Instead, grasses were grouped with the other core angiosperms with strong BS (86–100%). Interestingly, contrary to most published studies (see Background and Table 1), *Amborella *alone did not emerge as sister to all other angiosperms in any of these analyses. Most commonly (Figs. 7B,7C,7D), *Amborella *and *Nymphaea *together comprised the sister lineage to other angiosperms (with 66–100% BS), whereas an equal-rates ML analysis found *Nymphaea *deepest (albeit with low, 47% BS) and *Amborella *next deepest (Fig. 7A).

## Discussion

### The grasses-sister topology is an LBA artifact

That long branch attraction can be a serious problem in phylogenetic inference has long been known to the systematics community, ever since this phenomenon was first explored by Felsenstein [37]. Felsenstein described conditions of unequal evolutionary rates under which phylogenetic inference will result not only in an incorrect topology, but will converge asymptotically to the wrong phylogeny with increasing confidence as more data are added, ultimately producing 100% support for the wrong tree (hence, be *positively *misleading). Hendy and Penny [39] showed that this phenomenon can occur for parsimony even under equal evolutionary rates if taxa are insufficiently sampled along a branch, while Lockhart et al. [61] showed that an ML equal-rates model can incorrectly join long branches when there is rate heterogeneity across sites. In the case of DNA sequence data, due to the limited number of character states, taxa with the greatest sequence divergence are expected to be "attracted" to each other by chance alone if long and short branches are sufficiently different in length. With large amounts of data, this can result in spurious, yet strongly supported, relationships.

**Figure 8:**
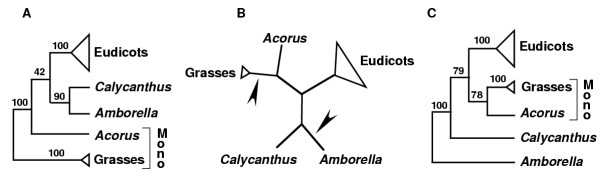
**Competing hypotheses for the rooting of angiosperms showing the same underlying angiosperm topology when outgroups are excluded. A**. Rooting within monocots (Mono), on the branch between grasses and all other angiosperms (see Fig. 2C, whose BS values are shown here, and also Fig. 2F; also see Goremykin et al. [19]). **B**. Unrooted network, with arrow showing alternative rootings as in A and C. **C**. Canonical rooting on the branch between *Amborella *and the rest of angiosperms (see Fig. 2I, whose BS values are shown here, and also Fig. 2L). We emphasize that 100% BS was obtained for *Amborella*-sister and for monocot monophyly (compared to 79% and 78% in C) using ML methods that allow for site-to-site rate heterogeneity (e.g., Additional files 1–3).

We used two complementary approaches to test the hypothesis that the grasses-sister topology favored in the study of Goremykin et al. [19] is caused by spurious attraction of the long branches leading to angiosperms and to grasses. Both approaches were designed to make the most direct comparisons possible to their dataset and phylogenetic methodology. First, and most importantly, we found that – even in the absence of corrections for rate heterogeneity – addition of just one more monocot to their dataset produced trees strongly supportive of 1) the *Amborella*-sister topology and 2) the idea that the grasses-sister topology is a consequence of LBA causing a misrooting of angiosperms. When the monocot *Acorus *was directly substituted for grasses, strong support for *Amborella*-sister was obtained (Fig. 2). This even occurred under analytical conditions that give strong support for grasses-sister when *Acorus *is not included. When *Acorus *and grasses were both included, two alternative, seemingly radically different topologies were obtained. Reconciliation of these topologies gets to the heart of the phylogenetic issues at hand. For as Fig. 8 shows, these two topologies are actually entirely congruent with respect to relationships among the various angiosperms, differing only in where the outgroup branch attaches within angiosperms [62], i.e., on the branches leading either to *Amborella *or to grasses (also see Fig. 5 and its treatment in Results).

The *Amborella*-sister topology is in agreement with the many diverse phylogenetic studies summarized in Table 1 and in Background, except for that of Goremykin et al. [19]. With *Acorus *included (Figs. 2I and 2L), it also shows monocots as monophyletic, consistent with a large body of evidence [7,35,41-43,63], and depicts faster chloroplast DNA evolution on the monocot lineage leading to grasses than in the *Acorus *lineage, also consistent with a substantial body of evidence (e.g. [9,44]). Conversely, the grasses-sister topology (Figs. 2C and 2F) is consistent only with the Goremykin et al. [19] results, fails to recover monophyly of monocots [has them either paraphyletic (Figs. 2C and 2F) or even polyphyletic (Fig. 3C), and always with 100% support], and fails to portray the known rapid evolution of chloroplast DNA in the lineages leading to grasses. All this leads us to conclude that the grasses-sister topology is almost certainly an artifact, most likely due to LBA between the long branches leading to grasses and to angiosperms.

Second, we reanalyzed the same dataset used by Goremykin et al. [19] and found that methods that account for rate heterogeneity across sites [61,64-67] put *Amborella *sister, usually with high BS (Figs. 2J, 3B, 4B, 4C, and 5; also see most Additional files). This was true for all 14 MODELTEST substitution models (Table 2) except for the simplest, JC model. When rates vary between sites, as with the chloroplast dataset under consideration, it is usually appropriate to model the evolutionary process to reflect this. The evolutionary models explored here point to LBA as the cause of the controversial grasses-sister topology and demonstrate that even with conservative corrections for rate heterogeneity, *Amborella *moves to the sister position within angiosperms (e.g., Figs. 5A and 5B).

In summary, our two principal approaches for reassessing the results and analyses of Goremykin et al. [19] lead to what we regard as compelling evidence for two major conclusions. First, *Amborella*, not grasses, is the sister angiosperm among this set of taxa. Second, any tendency for angiosperms to root on grasses is an LBA artifact stemming from the confluence of limited taxon sampling, rapid evolution in grasses, a long branch between the outgroups and angiosperms, and rate heterogeneity across sites. Furthermore, we point out that while our manuscript was nearly finished, two independent papers appeared [68,69] that also challenged Goremykin et al. [19] and reached similar conclusions to our study. Both studies are complementary to ours, because instead of taking the Goremykin et al. [19] 61-gene chloroplast dataset as the starting point, as we did, they used a 3-gene dataset (the same two chloroplast genes and one nuclear gene) plus the Goremykin et al. [19] set of taxa as the starting point for a variety of taxon-sampling experiments. In addition, an important forthcoming study [70] which added five new chloroplast genome sequences to the dataset of Goremykin et al. [19], found "strong support" for the Amborella-sister topology. That four entirely independent studies, using a variety of taxon sets, character sets, and analytical approaches, all lead to such similar results and conclusions makes it all the more likely that the grasses-sister topology is indeed a phylogenetic artifact.

### Is *Amborella *or *Amborella*+Nymphaeaceae sister to the rest of angiosperms?

Although our results reject grasses/monocots as the sister to all other angiosperms, support for *Amborella *as the first branch of angiosperm evolution must necessarily be qualified given the very limited sampling of whole chloroplast genomes (besides *Amborella*, only monocots, *Calycanthus*, and eudicots; see Fig. 1). There is still uncertainty as to the exact placement of *Amborella *relative to the other two deepest lineages of angiosperms, especially Nymphaeaceae [8,9], although the overall weight of published evidence currently favors *Amborella *as *the *deepest angiosperm (see [10,12] and references in Table 1). This uncertainty is heightened by our limited analyses that included *Nymphaea *and used methods that account for rate heterogeneity. These analyses never recovered an *Amborella*-sister topology. Instead, they most commonly found a sister clade comprising both *Amborella *and *Nymphaea *(Figs. 7B,7C,7D), or even found *Nymphaea *alone to be the sister-most angiosperm (Fig. 7A). Likewise, in the one analysis reported by Goremykin et al. [40] in which *Amborella *and *Nymphaea *were found sister to the other angiosperms these two taxa clustered as sisters rather than forming a basal grade.

Clearly, then, the question of which group is sister to the rest of extant angiosperms should be regarded as unsettled and in need of further exploration, using much more data (such as whole chloroplast genomes from a large number of diverse angiosperms, as well as more mitochondrial and/or nuclear data) and better analytical methodologies as they become available. At the same time, we must face up to two serious limitations arising from extinction. First, *Amborella trichopoda *is the only known species in the entire Amborellaceae/Amborellales, i.e., it is the only taxon available whose DNA can be used to represent a lineage of ca. 150 million years in age arising at or near the base of angiosperms. Second, the stem branch leading to angiosperms is long in length and years [9,62] (also approaching 150 million years) and thus represents a long-branch attractor, with the potential to spuriously attract other branches besides that leading to grasses. LBA between outgroup and ingroups is particularly insidious, because, as illustrated in Fig. 2 (C and F vs. I and L), it tends to mask the long nature of the ingroup branches. *Amborella *does not show any evidence of having a long branch in published analyses with more extensive taxon sampling. It is nonetheless difficult to rule out (but see [10]) the possibility that *Amborella *may be only near-sister among angiosperms (e.g., part of a Nymphaeaceae/*Amborella *clade that itself is the earliest branch of angiosperms; as suggested by Barkman et al. [8] and some of our analyses), with its generally sister position representing only a slight topological distortion (nearest neighbor interchange) caused by attraction to the long outgroup branch. For that matter, we point out (also see [71]) that the long branch leading to angiosperms also makes it difficult to rule out the possibility that the monophyletic-gymnosperm topologies recovered by multigene analyses (e.g., [35,72-74]) might result from LBA between angiosperms and the outgroup branch leading to seed plants.

### General implications

Many of our analyses, including all but one of the 61-gene concatenate analyses shown, included only first and second codon positions. This is because Goremykin et al. [19] chose to exclude third codon positions from their analyses, and because we wanted to make the most direct comparisons possible to their analyses. Third positions were excluded because most of the 61 chloroplast genes were claimed to be "very divergent" at synonymous sites (K_s _for most genes between *Pinus *and angiosperms was between 0.50 and 1.50 substitutions/site), which they felt could lead to "misleading" phylogenetic results. However, because our analyses with all three positions or only third positions gave such similar results to those using only first and second positions, we believe that for this particular dataset third positions are not contributing "excessive" homoplasy and leading to spurious affiliations. This conclusion is consistent with a considerable body of literature dealing with the phylogenetic utility of third positions in organellar genes [75-80], while simulations have shown that "saturated" data can be very reliable, provided that taxon sampling is sufficiently high [21,24]. Caution is nonetheless well advised in situations involving relatively sparse taxon sampling (some of which may be unavoidable, i.e., where extinction has been significant) and/or greater divergences than in this study. For example, chloroplast third positions are problematic in analyses across all of algal/plant evolution (e.g., [81]), and even appear to be problematic at the relatively shallow level of seed plant phylogeny [35,73,82].

Our findings, and those of others [68-70,83], highlight the potential danger of phylogenetic analyses that employ lots of genes, but too few and/or the wrong taxa. Adequate taxon sampling is in a sense even more important here than with single or few-gene trees, because of the potential for even subtle systematic bias in a particular lineage's evolution to generate strongly supported misleading trees. Equally, if not more importantly, our results emphasize the crucial importance of using phylogenetic methods that best model the underlying molecular evolutionary processes, especially by accounting for site-to-site rate variation.

## Methods

### Sequencing chloroplast genes from *Acorus*

We used long PCR to generate full-length or partial sequences from *Acorus gramineus *Soland. (a voucher specimen is deposited at the IND herbarium) for 22 of the 61 chloroplast genes analyzed by Goremykin et al. [19]. Long PCRs were conducted using the AccuTaq™ LA DNA Polymerase (Sigma, Atlanta, GA, USA), following instructions provided by the manufacturer. Initially, sets of primers designed by Graham and Olmstead [9], which cover a large portion of the chloroplast genome (*psbC-D *and *psbE-J *operons; from *rpl2 *to 3'-*rps12 *gene), as well as the primers described in [84-87] for the *rbcL*, *atpB*, *trnL-F*, and *trnE-D *region, respectively, were used for amplifications and/or sequencing. For the most part, however, based on the initial sequences, a number of sequencing primers were designed and used for chromosome walking with long PCR products. Primer sequences are available upon request from SS. PCR products were separated by electrophoresis using 0.8% agarose gels, visualized with ethidium-bromide, and cleaned using Qiagen columns (Valencia, CA, USA). Cleaned products were then directly sequenced using the BigDye™ Terminator cycle sequencing kit (PE Applied Biosystem, Foster City, CA, USA) on an ABI 3100 DNA automated sequencer (PE Applied Biosystem, Foster City, CA, USA). Sequence data were edited and assembled using Sequencher™ 4.1 (Gene Codes Corporation, Ann Arbor, MI, USA). The *Acorus *sequences for these 22 chloroplast genes (*atpA*, *atpE*, *clpP*, *cemA*, *lhbA*, 3'-*petB*, *petD*, *petG*, *petL*, *psaB*, *psaI*, *rpl20*, *rpoA*, *rpoB*, *rpoC1*, *rpoC2*, *rps2*, *rps14*, *rps18*, *rps19*, *ycf3*, *ycf4*) are deposited in GenBank (accession numbers AY757810-AY757831). These were combined for phylogenetic analyses with full-length or partial *Acorus *sequences already available in GenBank for 18 other chloroplast genes [AF123843 (*psbB*, *psbT*, *psbN*, *psbH*), AF123771 (*rps7*, 3'-*rps12*), AF123828 (*psbE*, *psbF*, *psbL*), AF123813 (*psbD*, *psbC*), AF123785 (*rpl2*), D28866 (*rbcL*), X84107 (*rps4*), U96631 (*psbA*), AB040155 (*matK*), AF197616 (*atpB*), and AJ344261 (*psaA*)]. The 40 *Acorus *genes used here come from two closely related species – *A. calamus *(14 genes) and *A. gramineus *(26 genes) – and correspond to 65.6% (40/61) of the genes and 71.4% (32,072/44,937) of the nucleotide characters analyzed by Goremykin et al. [19].

### Alignment

For all first and second codon position analyses, the data matrix provided by V. Goremykin was used without modification. For analyses that included *Acorus*, the *Acorus *genes were individually aligned with the individually extracted gene alignments from the Goremykin et al. [19] dataset using CLUSTALW [88], and the resulting gene alignments were concatenated to regenerate a matrix identical to the original except for the extra row containing *Acorus*. Using the same procedure, *Acorus *was also added to the amino acid matrix provided by V. Goremykin. The relevant 61 chloroplast genes of *Nymphaea *[40] were likewise added to both alignments.

We also constructed a new matrix consisting of all three codon positions by extracting genes from 13 sequenced chloroplast genomes of land plants (GenBank numbers: AP002983, AP000423, AJ271079, Z00044, AJ400848, AJ506156, AJ428413, X86563, AB042240, X15901, D17510, AP004638, X04465), aligning them, and hand editing apparent mistakes. The first and second position version of this matrix was nearly identical to the Goremykin et al. [19] matrix, except for a few minor differences (the overall length was slightly shorter due to removal of terminal extensions that either were created by single taxon indels or where multiple extending genes were nonhomologous). All phylogenetic trees resulting from this first and second position matrix and the Goremykin et al. [19] matrix were identical in topology and nearly identical in BS values. All alignments used in this study are available in Nexus format upon request of DWR.

### Phylogenetic analyses

Phylogenetic analyses were performed in PAUP* 4.0b10 [45]. Unless specified, all nucleotide-based trees were built using only first- and second-codon positions. For ML analyses, parameters were initially estimated using an equal-weighted parsimony tree. A ML tree was then built, and parameters were re-estimated using this tree if it differed from the parsimony tree. This iteration was continued until the last two topologies converged (the final ML topology was almost always equal to the one in which the ML parameters were estimated from the parsimony topology). For all ML analyses we also calculated a NJ tree using distances calculated from the ML model being tested. For DNA and protein parsimony the default PAUP* 4.0b10 [45] step matrices were used.

Bootstrap support [89] was estimated with 100 replicates using parameters estimated from the final topology. Thus the methodology cited for a particular tree refers to the model used for the bootstrap replicates. For parsimony and ML searches the heuristic algorithm was used with simple and as-is stepwise addition, respectively; tree bisection-reconnection swapping; and no limit on the number of trees saved in memory. Unless specified, the default PAUP* settings were used in all analyses. An automated script (available upon request from DWR) was used to run the analyses. Detailed log files and trees of each analysis were saved and are available upon request from DWR. Most analyses were performed on two 3 GHz Linux machines. Treetool [90] was used for viewing and printing trees.

The Shimodaira-Hasegawa (SH) test [59] was performed using the "lscores" command of PAUP* with the options SHTest = RELL and BootReps = 10000. ML parameters being tested were estimated on each topology to calculate its own log likelihood except where otherwise specified.

## Abbreviations

BS – bootstrap support; LBA – long branch attraction; ML – maximum likelihood; MP – maximum parsimony; NJ – neighbor joining; Ti/Tv – transition:transversion; NT – nucleotides; Plnvar – proportion of invariant sites

## Authors' contributions

SS generated the new sequences (from *Acorus*) used in this study and conceived and drafted the first and last figures. DWR carried out the phylogenetic analyses and made all other figures. All three authors contributed to the overall design of the study, drafted parts of the manuscript, and read and approved the final manuscript.

## Supplementary Material

Additional File 1 **Trees from truncated matrix with *Acorus***. These first- and second-position trees show that the results are essentially the same when positions that have *Acorus *data missing are removed. The first row using the ML HKY85 model is with four rate categories and parameters estimated as described in Methods. The third row uses the ML model parameters calculated as in the first row to calculate a distance matrix that was used for NJ analyses. For comparison the corresponding bootstrap values for *Amborella *sister to the angiosperms in the full matrix, going across each row, are 1. (99 vs. 100, 100 vs. 100), 2. (NA but same topology and similar BS, 100 vs. 100), 3. (86 vs. 88, 84 vs. 90). Click here for file

Additional File 2 **Trees from truncated RY-coded matrix with *Acorus *included**. This are the same analyses as in Additional file 1 except the DNA is RY-coded. For comparison, the corresponding BS values for the *Amborella *sister relationship in the full matrix, along each row, are: 1. (100 vs. 100, 100 vs. 100), 2 (98 vs. 100, 100 vs. 100), 3. (100 vs. 100, 100 vs. 100). Click here for file

Additional File 3 **Comparison of gamma-distributed rates with two versus four rate categories**. This figure shows that using two rate categories gives essentially the same results as using four rate categories with this dataset. The dataset is the first- and second-position, 61-gene matrix with grasses, *Acorus*, or both used to represent monocots. The ML HKY85 model was used and parameters were estimated as described in Methods. Click here for file

Additional File 4 **Trees when constant sites are removed from the first- and second-position matrix of Goremykin et al. [19]**. **A**. ML HKY85 and equal rates. **B**. NJ with distances calculated using an ML HKY85 model and equal rates. Click here for file

Additional File 5 **NJ analysis using ML proportion of invariant distances**. Distances were calculated using the ML HKY85 model, the estimated proportion of invariant sites, and the first- and second-position matrix of Goremykin et al. [19]. Click here for file

Additional File 6 **ML trees using third positions only**. **A**. HKY85 model with equal rates. **B**. HKY85 model with four gamma-distributed rates. Click here for file

Additional File 7 **Sister group to the rest of angiosperms found in individual gene analyses using first- and second-position data without *Acorus *Top**, ML HKY85 with four gamma-distributed rates. **Bottom**, Parsimony analysis. Click here for file

Additional File 8 **Sister group to the rest of angiosperms found in individual gene analyses using the ML HKY85 model with four gamma-distributed rates and *Acorus *added**. **Top**, all three positions. **Bottom**, first and second positions. Click here for file

Additional File 9 **Sister group to the rest of angiosperms found in individual gene analyses using the ML HKY85 model with four gamma-distributed rates with *Acorus *added and grasses removed**. **Top**, all three positions. **Bottom**, first and second positions. Click here for file

Additional File 10 **Sister group to the rest of angiosperms found in individual gene analyses using parsimony on all three positions**. **Top**, *Acorus *added. **Bottom**, *Acorus *added and grasses excluded. Click here for file
