# Supplementary material for: Long branch attraction, taxon sampling, and the earliest angiosperms: Amborella or monocots?
Source: BMC Evol Biol. 2004 Sep 28;4:35. doi: 10.1186/1471-2148-4-35 (PMC543456; doi:10.1186/1471-2148-4-35)
Supplement: Additional File 1 — Trees from truncated matrix with Acorus. These first- and second-position trees show that the results are essentially the same when positions that have Acorus data missing are removed. The first row using the ML HKY85 model is with four rate categories and parameters estimated as described in Methods. The third row uses the ML model parameters calculated as in the first row to calculate a distance matrix that was used for NJ analyses. For comparison the corresponding bootstrap values for Amborella sister to the angiosperms in the full matrix, going across each row, are 1. (99 vs. 100, 100 vs. 100), 2. (NA but same topology and similar BS, 100 vs. 100), 3. (86 vs. 88, 84 vs. 90). [file 1471-2148-4-35-S1.pdf]

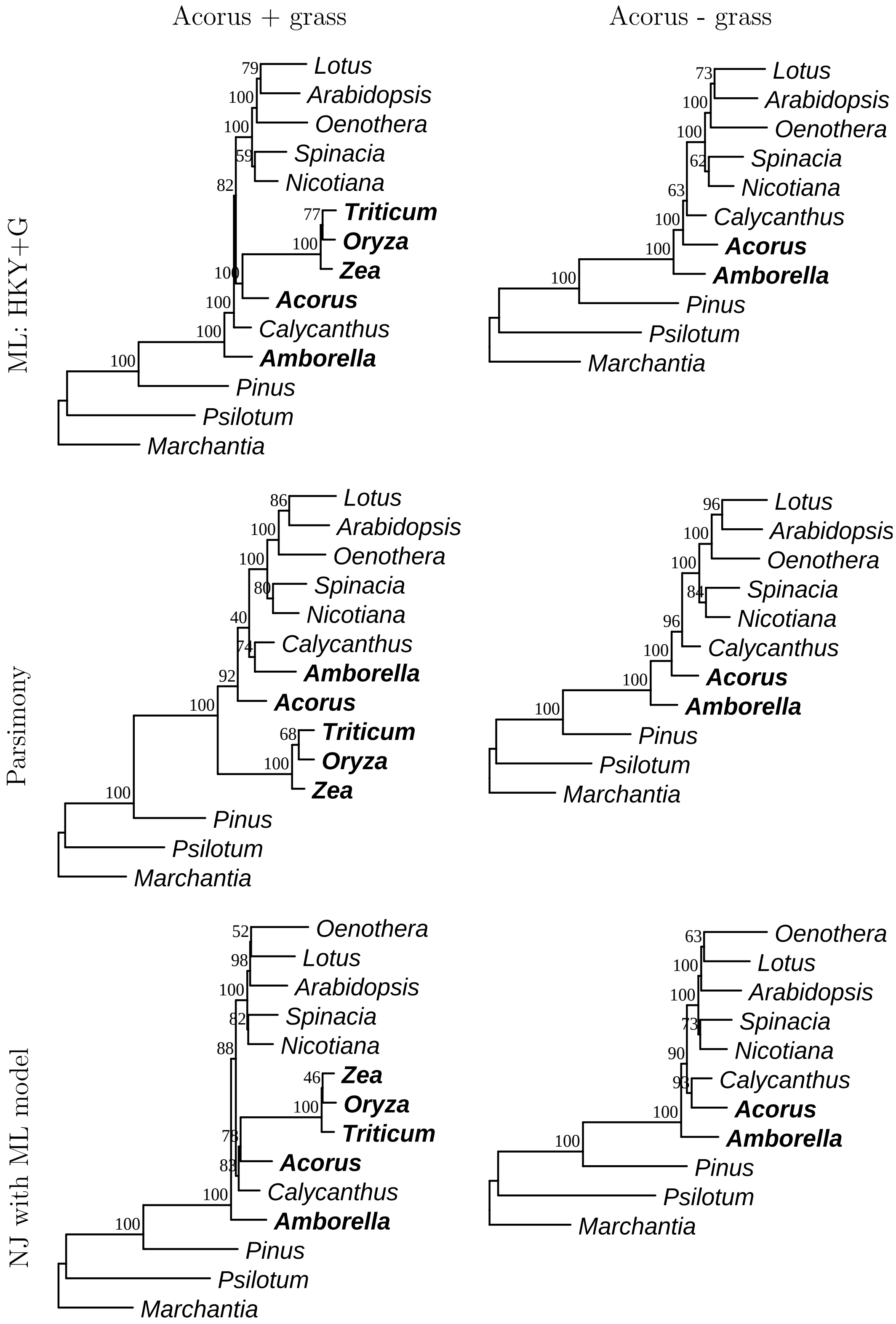

Trees from truncated matrix with *Acorus*: These trees show that the results are esssentially the same when positions that have *Acorus* data missing are removed. The first row using the ML HKY85 model is with 4 rate categories and parameters estimated as described in Methods. The third row uses the ML model parameters calculated as in the first row to calculate a distance matrix to be used with NJ. For comparison the corresponding bootstrap values for *Amborella* sister to the angiosperms in the full matrix, going across each row, are 1. (99 vs. 100, 100 vs. 100) 2. (NA but same topology and similar BS, 100 vs. 100) 3. (86 vs. 88, 84 vs. 90).
