# Supplementary material for: Long branch attraction, taxon sampling, and the earliest angiosperms: Amborella or monocots?
Source: BMC Evol Biol. 2004 Sep 28;4:35. doi: 10.1186/1471-2148-4-35 (PMC543456; doi:10.1186/1471-2148-4-35)
Supplement: Additional File 2 — Trees from truncated RY-coded matrix with Acorus included. This are the same analyses as in Additional file 1 except the DNA is RY-coded. For comparison, the corresponding BS values for the Amborella sister relationship in the full matrix, along each row, are: 1. (100 vs. 100, 100 vs. 100), 2 (98 vs. 100, 100 vs. 100), 3. (100 vs. 100, 100 vs. 100). [file 1471-2148-4-35-S2.pdf]

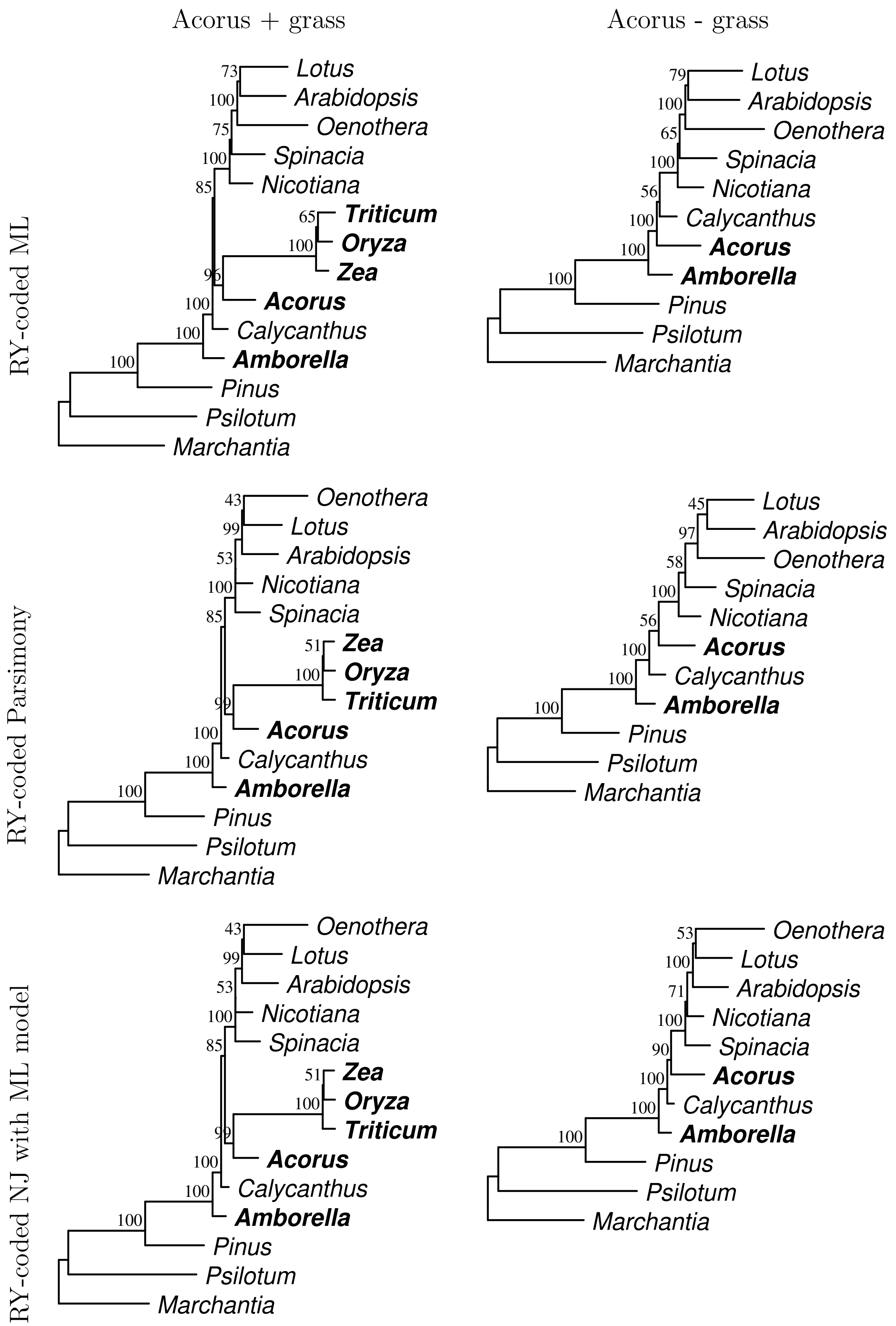

Trees from truncated RY-coded matrix with *Acorus* included This is the same analysis as in Additional file 1 except the DNA is RY-coded. For comparison, the corresponding BS values for the Amborella sister relationship in the full matrix, along each row, are: 1. (100 vs. 100, 100 vs. 100) 2. (98 vs. 100, 100 vs. 100) 3. (100 vs. 100, 100 vs. 100).
