# Supplementary material for: Long branch attraction, taxon sampling, and the earliest angiosperms: Amborella or monocots?
Source: BMC Evol Biol. 2004 Sep 28;4:35. doi: 10.1186/1471-2148-4-35 (PMC543456; doi:10.1186/1471-2148-4-35)
Supplement: Additional File 3 — Comparison of gamma-distributed rates with two versus four rate categories. This figure shows that using two rate categories gives essentially the same results as using four rate categories with this dataset. The dataset is the first- and second-position, 61-gene matrix with grasses, Acorus, or both used to represent monocots. The ML HKY85 model was used and parameters were estimated as described in Methods. [file 1471-2148-4-35-S3.pdf]

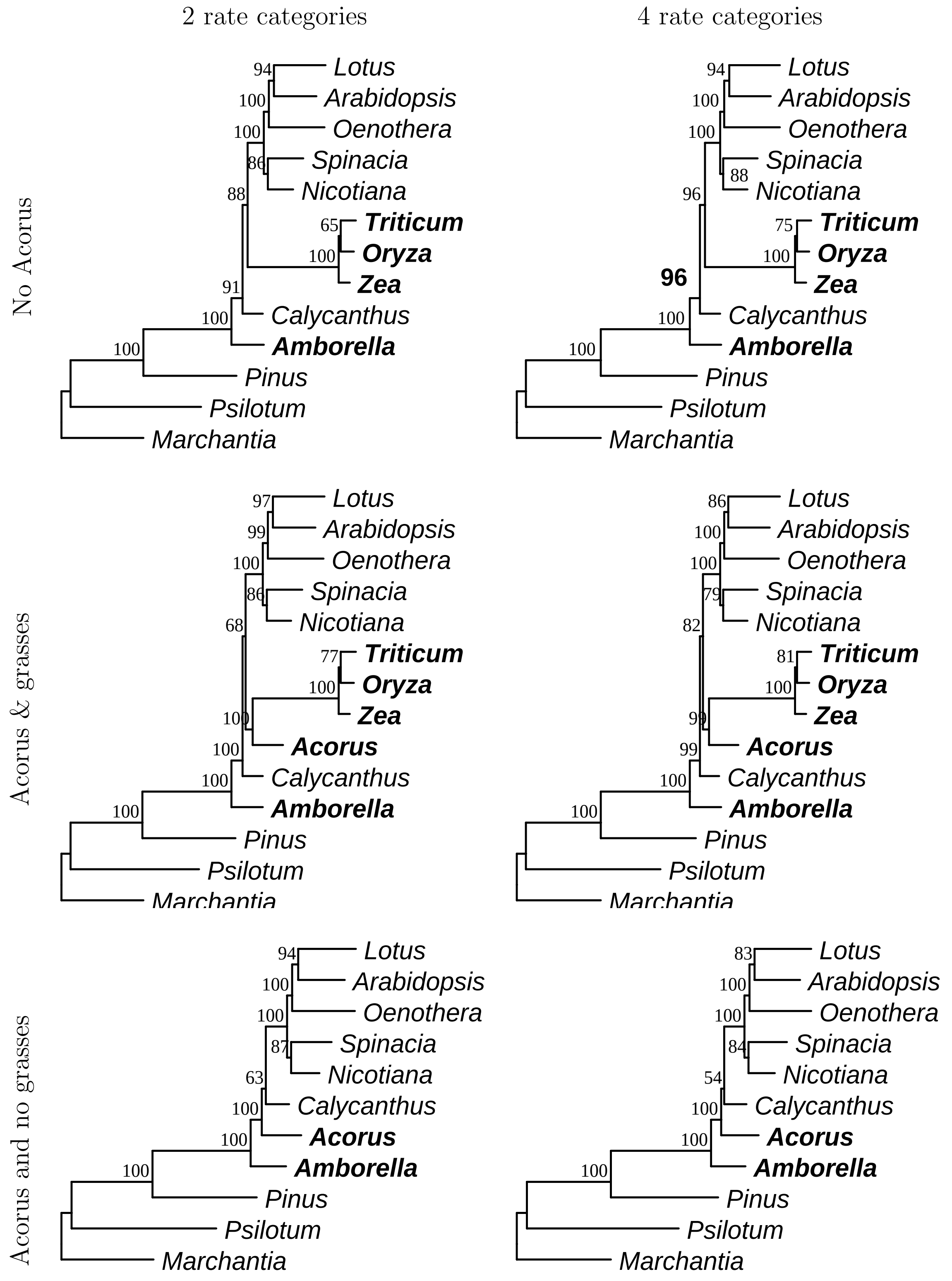

Comparison of gamma distributed rates with 2 versus 4 rate categories: This figure shows that using two rate categories gives essentially the same results as using four rate categories with this dataset. The dataset is the first- and second-position 61 gene matrix with either grasses, *Acorus*, or both used to represent monocots. The ML HKY85 model was used and parameters were estimated as described in Methods.
