# Supplementary material for: Long branch attraction, taxon sampling, and the earliest angiosperms: Amborella or monocots?
Source: BMC Evol Biol. 2004 Sep 28;4:35. doi: 10.1186/1471-2148-4-35 (PMC543456; doi:10.1186/1471-2148-4-35)
Supplement: Additional File 4 — Trees when constant sites are removed from the first- and second-position matrix of Goremykin et al. [19]. A. ML HKY85 and equal rates. B. NJ with distances calculated using an ML HKY85 model and equal rates. [file 1471-2148-4-35-S4.pdf]

A B

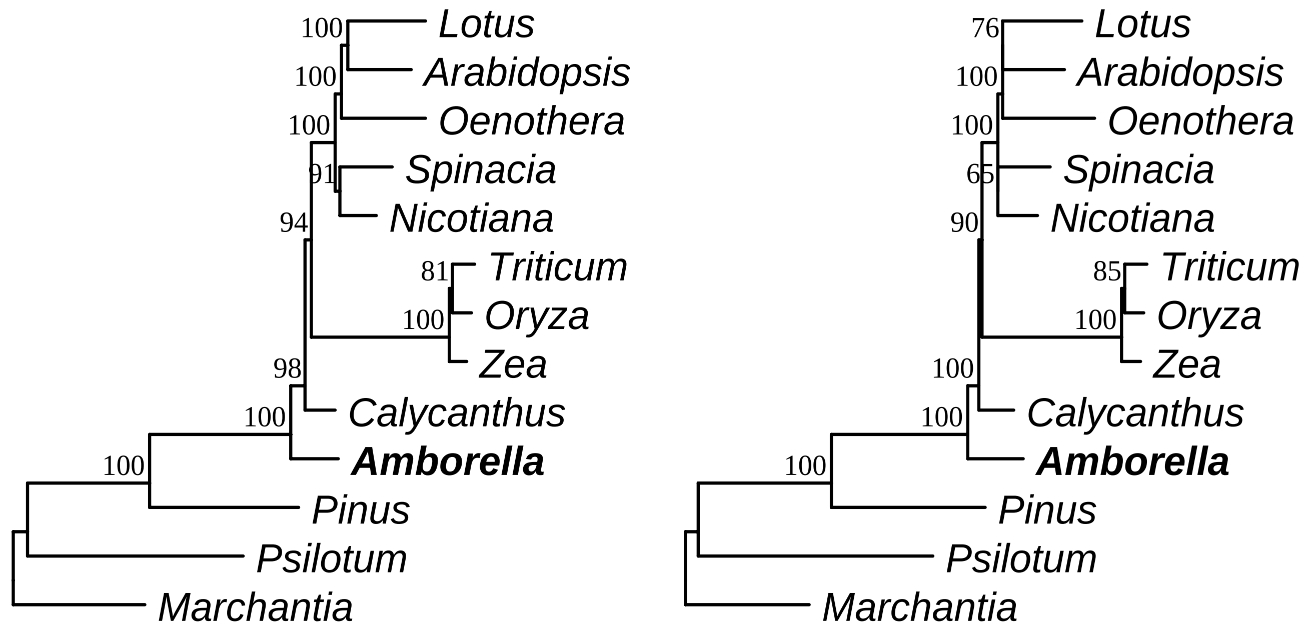

Trees when constant sites are removed from the 1st and 2nd position alignment of Goremykin et al. 2003

A. ML HKY85 and equal rates.
B. NJ with distances calculated using an ML HKY85 model and equal rates
