# Supplementary material for: Long branch attraction, taxon sampling, and the earliest angiosperms: Amborella or monocots?
Source: BMC Evol Biol. 2004 Sep 28;4:35. doi: 10.1186/1471-2148-4-35 (PMC543456; doi:10.1186/1471-2148-4-35)
Supplement: Additional File 5 — NJ analysis using ML proportion of invariant distances. Distances were calculated using the ML HKY85 model, the estimated proportion of invariant sites, and the first- and second-position matrix of Goremykin et al. [19]. [file 1471-2148-4-35-S5.pdf]

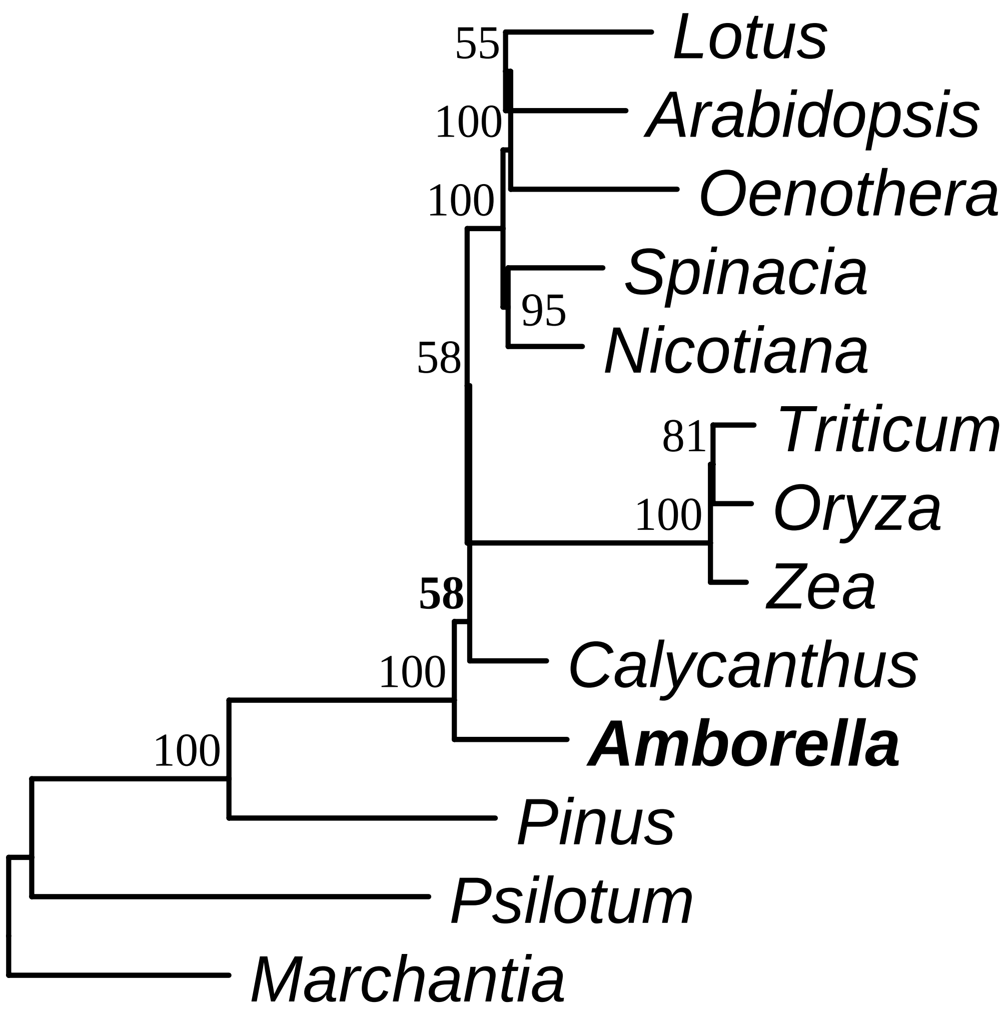

NJ analysis with ML proportion invariant distances Distances were calulated using the ML HKY85 model and estimated proportion of invariant sites. The first- and second-position matrix of Goremykin et al. 2003.
