# Supplementary material for: Long branch attraction, taxon sampling, and the earliest angiosperms: Amborella or monocots?
Source: BMC Evol Biol. 2004 Sep 28;4:35. doi: 10.1186/1471-2148-4-35 (PMC543456; doi:10.1186/1471-2148-4-35)
Supplement: Additional File 6 — ML trees using third positions only. A. HKY85 model with equal rates. B. HKY85 model with four gamma-distributed rates. [file 1471-2148-4-35-S6.pdf]

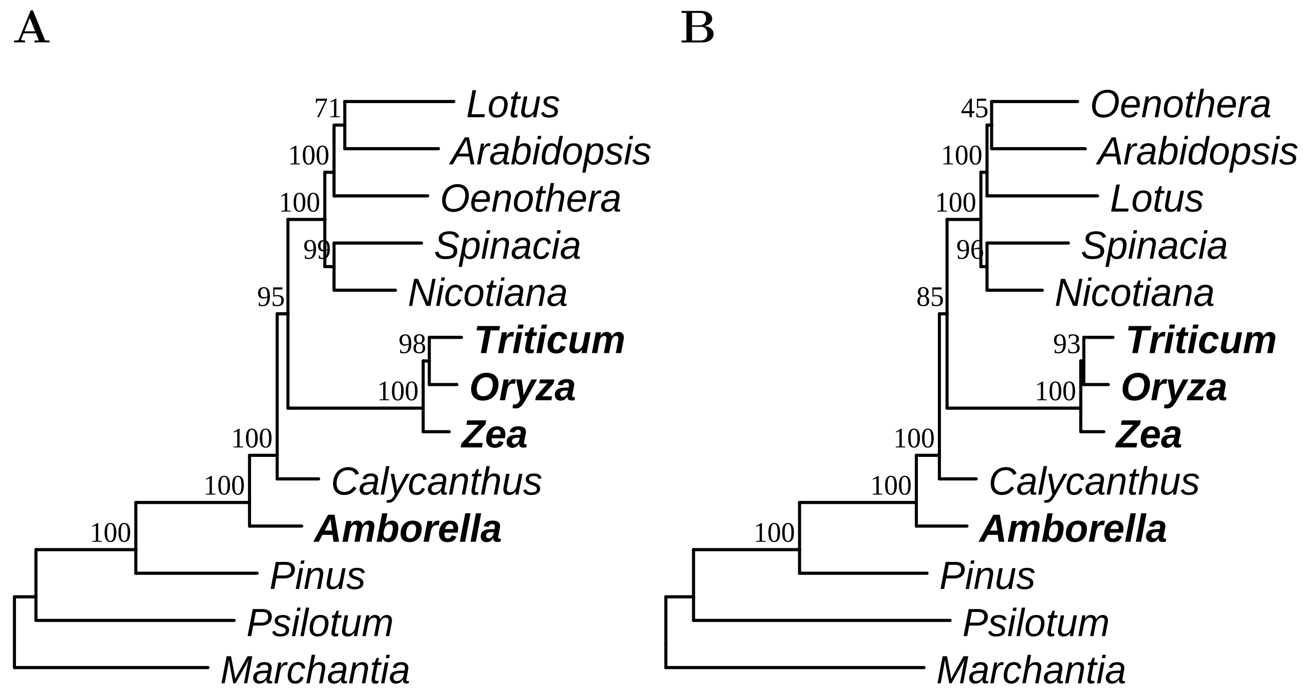

ML trees using third positions only

A. HKY85 model with equal rates.
B. HKY85 model with gamma rates and 4 rate categories
