# Supplementary material for: Long branch attraction, taxon sampling, and the earliest angiosperms: Amborella or monocots?
Source: BMC Evol Biol. 2004 Sep 28;4:35. doi: 10.1186/1471-2148-4-35 (PMC543456; doi:10.1186/1471-2148-4-35)
Supplement: Additional File 7 — Sister group to the rest of angiosperms found in individual gene analyses using first- and second-position data without Acorus Top, ML HKY85 with four gamma-distributed rates. Bottom, Parsimony analysis. [file 1471-2148-4-35-S7.pdf]

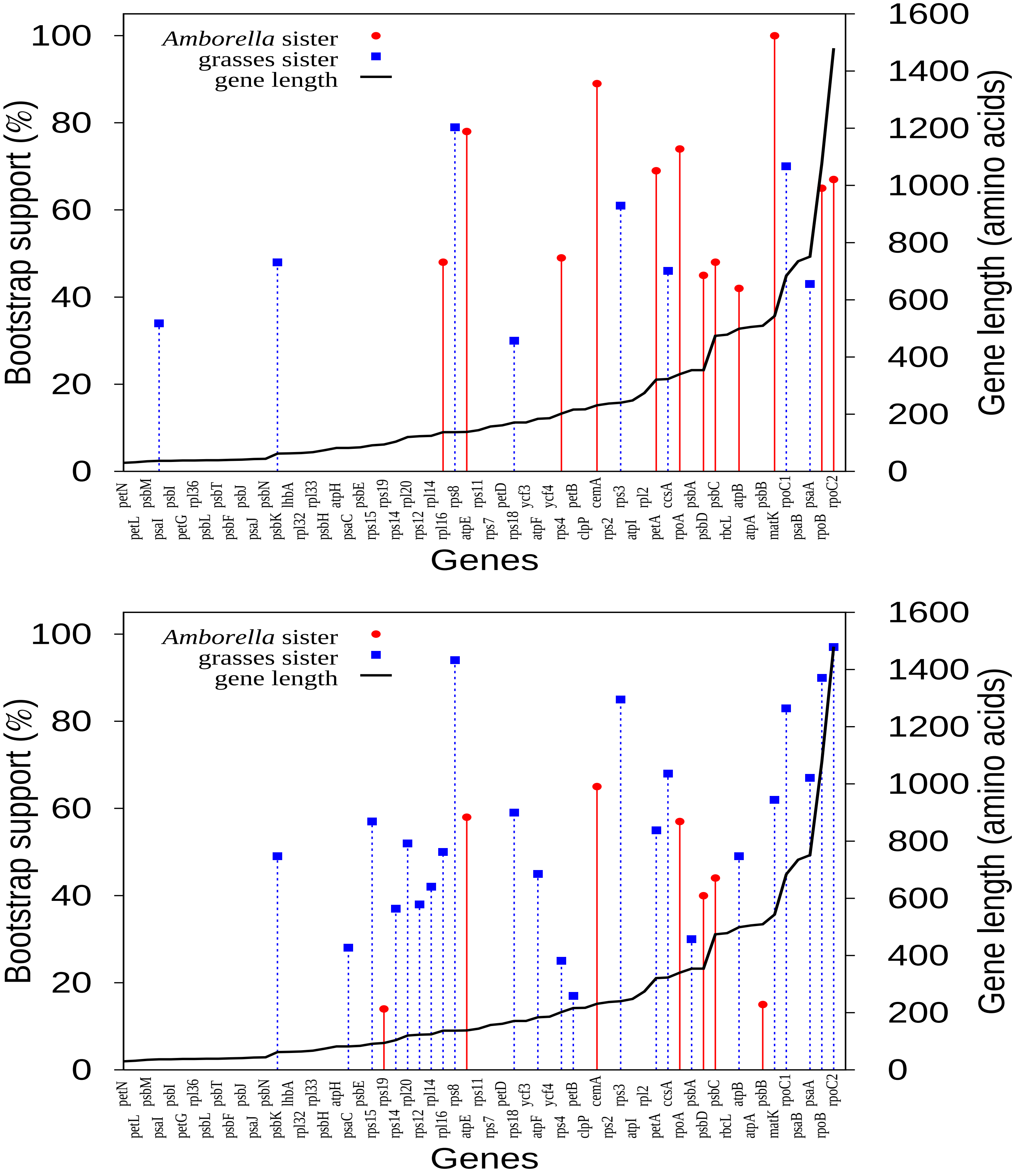

Sister group to the rest of angiosperms found in individual gene analyses using first- and second-position data without *Acorus*

Top: ML HKY85 with four gamma distributed rates
Bottom: Parsimony analysis
