# Supplementary material for: Long branch attraction, taxon sampling, and the earliest angiosperms: Amborella or monocots?
Source: BMC Evol Biol. 2004 Sep 28;4:35. doi: 10.1186/1471-2148-4-35 (PMC543456; doi:10.1186/1471-2148-4-35)
Supplement: Additional File 8 — Sister group to the rest of angiosperms found in individual gene analyses using the ML HKY85 model with four gamma-distributed rates and Acorus added. Top, all three positions. Bottom, first and second positions. [file 1471-2148-4-35-S8.pdf]

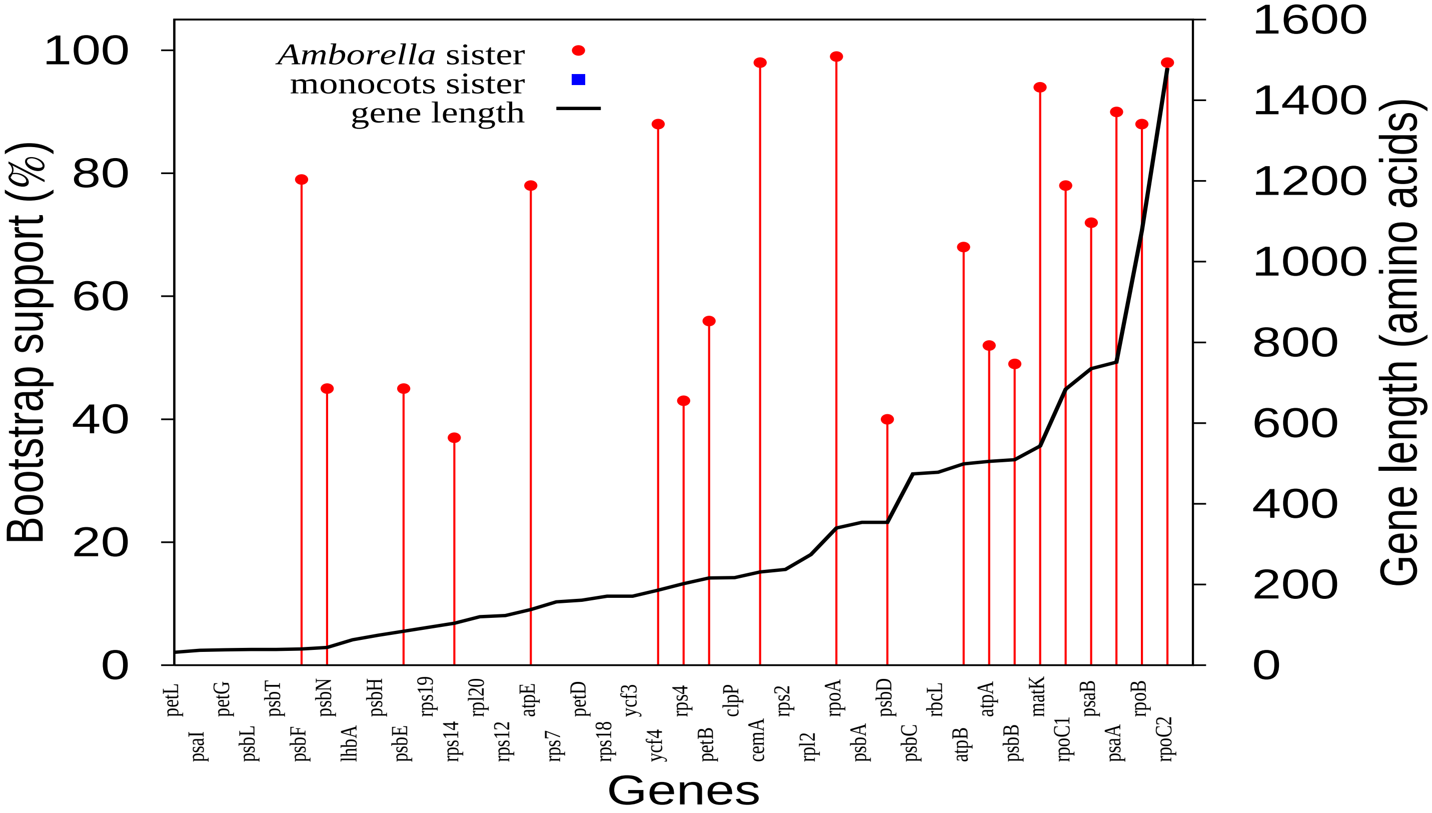

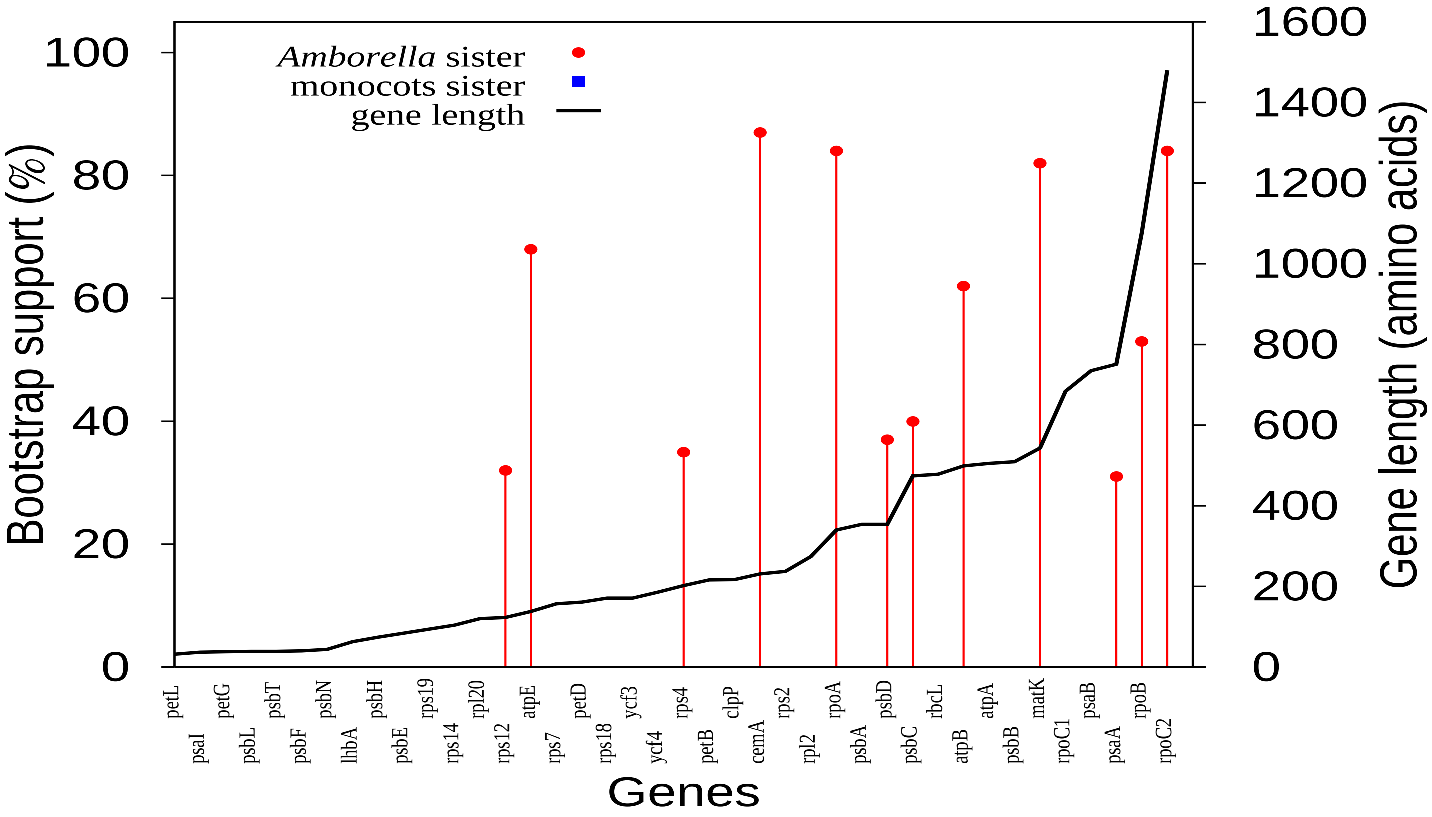

Sister group to the rest of angiosperms found in individual gene analyses using the ML HKY85 model with 4 gamma distributed rates and *Acorus* added

Top: all 3 positions
Bottom: 1st and 2nd positions
