# Supplementary material for: Long branch attraction, taxon sampling, and the earliest angiosperms: Amborella or monocots?
Source: BMC Evol Biol. 2004 Sep 28;4:35. doi: 10.1186/1471-2148-4-35 (PMC543456; doi:10.1186/1471-2148-4-35)
Supplement: Additional File 10 — Sister group to the rest of angiosperms found in individual gene analyses using parsimony on all three positions. Top, Acorus added. Bottom, Acorus added and grasses excluded. [file 1471-2148-4-35-S10.pdf]

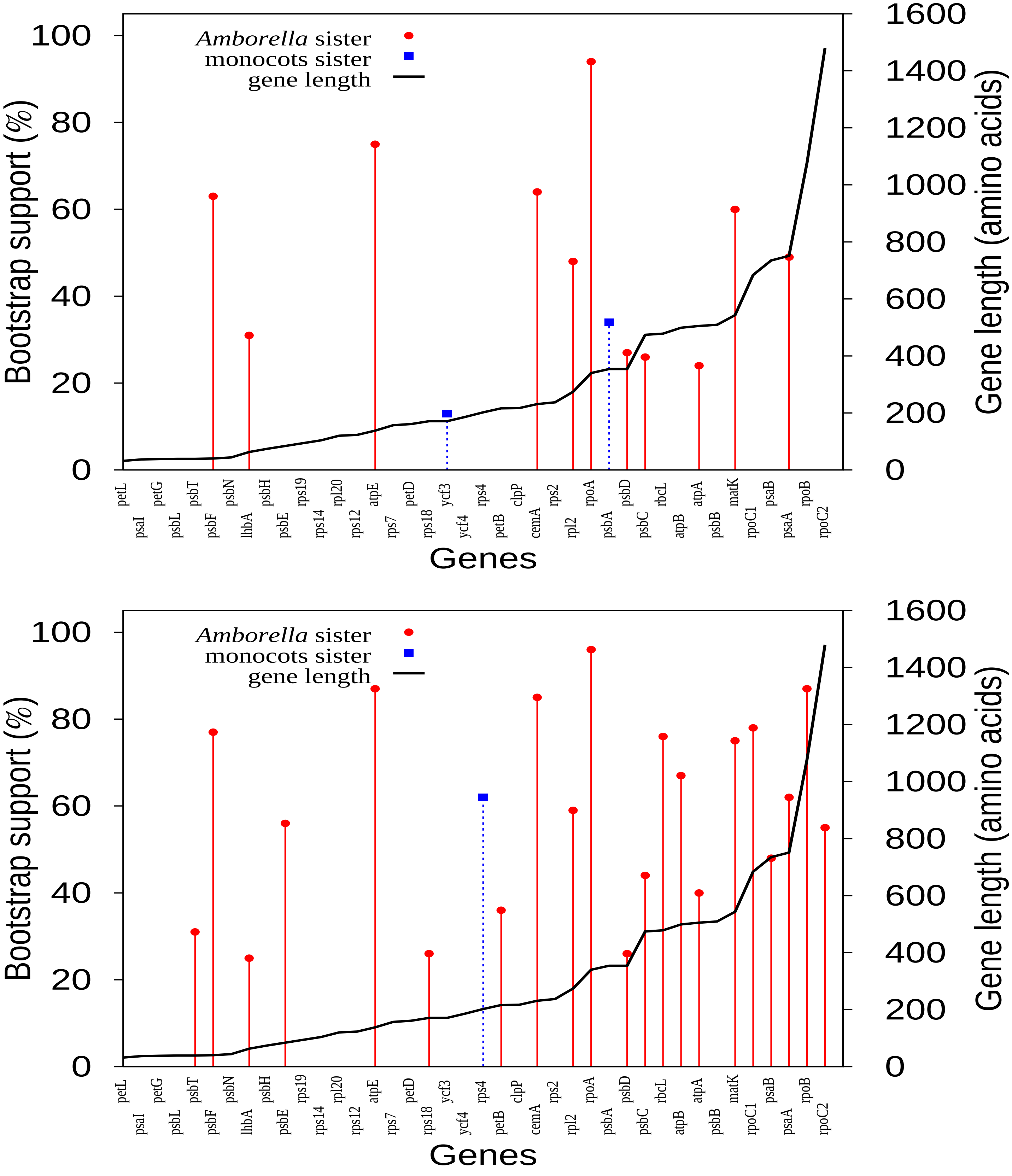

Sister group to the rest of angiosperms found in individual gene analyses using parsimony on all 3 positions

Top: *Acorus* added
Bottom: *Acorus* added minus grasses
